# Repertory of the Back

**Published:** 1897-10

**Authors:** E. H. Wilsey

**Affiliations:** Parkersburg, W. Va.


### REPERTORY OF THE BACK.

DR. E. H. WILSEY, PARKERSBURG, W. VA.

**Abdomen.** Pain in back at night, allowing one only to lie on abdomen, NIT-A.

— frequent chilliness on arms, back, and abdomen, Sarsa.

— puffed with backache, Cimex.

— first some chilliness down the back with icy cold hands, then intense heat with distention of abdomen, Sil.

— cramps in muscles of back and abdomen on turning head and body, Dros.

— cutting in back and abdomen extending through urethra, Canth.

— a violent stitch above within the right ribs darting out at the abdomen and back, Nit-a.

— during second day of menses coryza, pain in abdomen, toothache, backache, stitches in the ears and restless sleep, Kali-c.

— pain in small of back extending over abdomen with distention of abdomen, *Cimex*.

— pain in middle of back, as if some one was pinching with pincers, extending gradually toward abdomen, Cann-s.

— paralytic pain in small of back, better lying on abdomen, worse bending backward, Selen.

**Aching** of back in general: All-s., Ars., Ars-s-f., Aur-mur., Bapt., *Bism.*, *Bry.*, *Graph.*, Ind., *Puls.*, *Sep.*, Ruta, Cap., Canth., *Psor.*, *Eup-perf.*, *Gels.*, *Sul.*, *Sil.*, Petrol., Mag-c., *Kali-c.*, *Nux-v.*, *Nat-m.*, Bov., Lach., Kreos., Arn., *Kalm.*, *Lyc.*, Vacc., Amm-c., *Poly.*, *Can-i.*, *Arg-n.*, *Clem.*, *Curare*, *Cham.*, Calc-fl., *Nat-c.*, *Ipec.*, Kali-br., *Rhus*, Nux-m., Podo., *Bell.*, *Lil-tig.*, *Lyss.*, Lac-can., Med., Trill., Asa.,

*Tereb.*, *Æsc-h.*, Diosc., *Casc.*, Zing., Vib., Inul., Amm-m., Rumex, Ant-c., Lact-ac., Phyto., *Calc-a.*, *Kali-b.*, *Aloe*, Ptel., Merc-per., *Caust.*, Plat., Ver., Sul-a., Bar-c.

**Aching** in back before menses, Acon., Amm-m., Brom., 1 Calc-c., *Caust.*, *Gels.*, Hyper., Kali-c., 1 Lyc., 1 Mag-c., 1 Spong.

— in back during menses, Aloe, Bell., Calc-phos., *Ign.*, *Graph.*, *Bry.*, 1 Kali-c., 1 Phos., 1 Nux-v.

— and sore pain across small of back, *Lact-ac.*

— dull pain in back and hips, Poly.

— beating and throbbing in back, *Sil.*

— intolerable aching in back, *Calc-c.*

— dull distress in whole dorsal region at 6 P. M., Erig.

— intolerable aching soreness as if beaten in small of back, Eup-perf.

— in back extending into feet, Borax.

— dull tired in shoulders and across back, *Curare.*

— in small of back, Ustil.

— on waking, better on getting up, with disinclination to move, Lappa.

— in small of back, as if bruised or broken, Conval., *Graph.*

— while sitting, small of back aches, as if sitting bent, or as after stooping, *Rhus.*

— a tired aching in small of back, as after a long ride, but did not ride far, can sit in no position to relieve back, Calc-fl.

— bruised feeling in evenings; he cannot straighten his back, Psorin.

— dull aching in back every morning on waking, Ptelia.

— dull aching drawing pain in back between shoulders at 6 P. M., Zing.

— intense aching in back and limbs, extremities feel as if broken, *Eup-pur.*

— in small of back, worse by either coughing or laughing, Tell.

— a thrilling quivering about an inch to left of dorsal vertebræ, four inches below scapula in evening; on rising from reclining it changed into aching, later a crawling with a thrilling, Ver-vir.

**Aching** in small of back, as if bruised, with coryza, Cinnab.

— in back, especially at night, with great weakness of legs, *Arg-n.*, *Am-m.*

— in small of back, as after taking cold, Ars-hyd.

— aching in back, confining him to bed (with caries of sternum and cough, Sul. partly cured the caries, Psor., the cough and Ars-sul., the aching and remaining caries), Ars-sul.

— distress in back, bowels, and legs, with rumbling of wind, Ptelia.

— a weak, stiff aching in back, Med.

— in small of back, preventing sleep with orgasm of blood with bruised sensation, *Sul.*

— in back when driving in evening, Sul.

— tired feeling, and some burning in back and legs of women, *Pic-ac.*

— of back, as if it would break, *Bell.*, Nux-v.

— must sit up to turn over in bed on account of back aching, *Nux-v.*

— at 10 A. M. during menses, then weakness of thighs, Amm-m.

— in upper part of back, as if beaten, Anac.

— in back, with variola, Ant-cr.

— in small of back, with urging and burning in anus, Merc-peren.

— in back and down left side of hip, Kali-b.

— in back, as if broken, *Kali-c.*

— first low down, but passing off, he feels it higher up at 8 P. M., several times with nausea, Zing.

— a stitch in back, then backache, Caustic.

— severe all day, with numbness of hands, Lyss.

— after hard work, *Arn.*

**Aching** constant of several years' standing, Sul.
— with leucorrhœa, *Mur-ac.*
— violent after walking, *Nat-c.*
— violent during menstruation, Inula.
— with tired lameness of legs, *Kali-br.*
— very severe, Aur-mur.
— as if it would break, *Bell.*
— with chill, *Ant-tart.*
— worse from pressure, Verbasc.
— worse from trying to sit up, *Zinc.*
— as from weakness, better when sitting and leaning against something, Zing.
— excessive backache, *Psor.*
— when walking with stitches in sternum, Psor.
— severe, as if bruised, cannot straighten out, *Psor.*
— with constipation, Psor.
— with suppressed eruption, *Psor.*
— caused by an emission, *Ham.*
— severe, better from pressure and lying on it, *Nat-m.*
— by lying on back and worse by eating, *Nat-m.*
— severe, better by passing urine, *Lyco.*
— and headache, *Lyss.*
— paining severely, Kali-c.
— as if bruised or broken, *Graph.*
— and weakness, inclination to lie down, and distaste for business, *Casc.*
— constant and dull, *Æsc-h.*
— walking almost impossible, causes backache, Æsc-h.
— in back, with seminal emissions, *Kobalt.*
— pressing in back at lower border of scapula, Rumex.
— in back, worse when stooping and walking, and from coffee, *Cham.*
— in back, with stiffness after stooping, Bov.
— in back, worse at menstrual periods, which occur every two weeks, and are scanty, *Can-ind.*

**Aching** and soreness in back, with kidney affection, *Tereb.*
— in back, causes nausea while standing, *Sep.*
— pain, with lame sensation in small of back, *Hyper.*
— pain in small of back of women at change of life, *Hydras.*
— pain in small of back when stooping, Jug-cin.
— pain in lower part of back for three months, *Ox-ac.*
— dull pain in back, extending up to occiput, *Gels.*
— violent, long, lasting aching pain about last lumbar vertebræ, *Zinc.*
— pain in back, worse in lumbar region, extending around the waist, Vacc.
— pain through small of back, *Cocc-c.*
— pains in small of back down to thighs, Carbolic-a.
— during P. M., much pain and tired aching in lower part of back, with bodily restlessness, must walk about, Calc-fl.
— and cutting pain about the lower dorsal vertebræ, *Asclep.*
**Acid** causes pain in back, with yawning and stretching, as in fever, Lach.
**Acute** pain in back, gradually extending down into thighs with great torture, better by change of posture, Ox-ac.
— pain beneath left shoulder, Mez.
**Across** hips, in back, a pain lasting all day, Lyss.
**Acute,** darting, boring pain in back, of neuralgic nature, *Mag-phos.*
**Across,** pain in back across hips, *Sepia.*
**Ague,** pain in back, as in cold stages of ague, Gels.
**Air.** Burning in back while walking in open air, when he gets warm, Sil.
— drawing in back and lumbar muscles with lassitude on exercising in open air, Tereb.
— strong aversion to air if it is let under bedclothes; while turning over it makes him chilly, *Nux-v.*
— burning pressure in back, worse when walking in open air, Kali-c.

**Air.** Sensation as if cool air was blowing on back, *Camph.*, Benz-ac.

— diseases brought on by exposure of back to draft of air, *Sil.*

— chilliness in back, without thirst in open air, especially in a draft, Dulc.

**Alternating** pain in back, with tightness in chest, Sil.

— great wearisome pains in thighs, as if they would fall off, or as if tendons would tear off, alternating with pains in small of back; she knows not what to do for the pains the third day of menses, Amm-c.

**Anxiety** and restlessness with pain in back, Ars.

**Ants.** Sensation as from ants crawling in back, Graph.

**Apart.** Pain as if muscles of back were stretched apart, Amm-m.

**Arch.** Back spasmodically curved and bent backward like an arch, violent trembling motion, *Opi.*

— back bent backward like an arch, *Cic.*

**Arm.** Lying on his back with left arm over his head, a half conscious sleep, Dig.

— pain from small of back into arms, Rhod.

**Arise.** Spasmodic pain in small of back does not allow him to arise, *Sil.*

**Asleep.** Burning, drawing pain in small of back during sleep in morning; also a sensation of falling asleep in shoulder joint, disturbing sleep, and disappearing on awaking, Zinc.

— heaviness in back in morning, almost as if asleep, *Sep.*

— twitching in back when asleep, Amm-carb.

— a feeling of warmth in lower part of back and small of back, as if lumbar region was asleep, extending down into sacrum, hips and posterior portion of thigh, *Berb.*

**Awaking.** A sensation of heat down the back in the morning on awaking, Con.

— pain in small of back, worse when sitting, lying, and in morning when awaking, *Berb.*

**Bag-pipe.** Music in the ears like the far-off humming of a bag-pipe when lying in bed on back, Nat-c.

**Band.** As if a band passed through the small of back, and everything was constricted, taking away the breath, especially morning, *Puls.*

**Bandaged.** Small of back feels as if tightly bandaged, *Puls.*

**Bearing** down in small of back, as if menses would come on, *Apis.*

— down in back during menses, *Podo.*

— down in back, *Kali-c.*, Vib.

— down in paroxysms, *Sep.*

— down, with uterine hemorrhage, *Ham.*

**Beaten.** Pain in small of back, as if beaten while standing or walking, Zing.

— heat, pain, and beating in small of back, Lac-can.

— pain in back, shoulders, and abdomen, as if beaten, excited by motion, Stram.

— pain in back and lower limbs, as if beaten in morning on rising, with weariness, Stann.

— pain in back, as if sore and beaten, Sul-a.

— pain, as if beaten in small of back, Alum., *Sil.*

— pain in small of back while sitting, as if beaten, Ang.

— paralytic pain in small of back, as if beaten, *Nat-m.*

— severe pain in back at night, as if it were beaten to pieces, worse when moving, also when at rest, Mag-c.

— pain, as if beaten in back, and as if wearied by too great effort, Zinc.

— pain in small of back upon grasping it, as if it had been beaten, *Rhus.*

— extremely sore back, as if beaten in evening, Lyss.

— bruised, lame feeling in back, with stitches, *Nat-m.*

— lower part of back feels as if beaten, Zinc.

— disabled and stiff, as if beaten in back, Berb.

— upper part of back aches, and feels as if beaten, Anac.

— soreness of back, and especially back of neck, as if beaten, Ars-id.

**Beaten.** Aching and throbbing in back, *Sil.*

— or tearing pressure, with chilliness, changing to headache and heat in head, Sil.

— in the back, Sil.

— and pulsating in back, Bar-carb.

**Bed.** Back feels as if sprained in P. M., and evening in bed, *Sep.*

— sensation as if vertebræ were gliding over one another when turning in bed, Sul.

— itching on back and shoulders, as from crawling insects, worse evenings on going to bed, Osmium.

— gnawing in lower part of back, worse from going to bed, Naja.

— pain in back, as if he had been lying on too hard bed, Bar-c.

— great pain in small of back, worse in bed, and better by pressure, Rhus.

— stiffness now in back, now in hips, painful on turning over in bed, obliged to hold breath, Sul.

— pain in small of back when getting out of bed in morning, *Sil.*

— gets out of bed on account of pain in back at 4 A. M., better after rising and walking about, *Nux-v.*

— pain in back, with weakness in legs, gradually increasing until locomotion became extremely difficult, and he was compelled to stay in bed, Phos.

**Bedtime.** Pain in small of back, becoming intolerable near bedtime, Sinapis.

**Bellyache.** Drawing in back, as if bruised through the spine, extending to sacrum and abdomen when much flatus accumulated with bellyache, and as the flatus was discharged leucorrhœa appeared, Caustic.

**Bend.** Pain in small of back, could not walk erect, must bend over, *Sul.*

— constant pain in small of back, obliging him to bend the back inward, Sabina.

**Bending.** Pain in small of back on bending backward, Mang.

— pain back sometimes, better by bending forward at other times bending backward, Plumb.

— muscles of back feel too short when bending forward, Agari.

— drawing in back ceasing on bending backward, Petrol.

**Bent.** Pressure in back opposite bowels, then oppressive stitches on least motion or breathing, so that he had to walk bent, when lying still; griping as in malignant ulcer, and with oppression of chest, Arg-m.

— often in circular form, Opi.

— backward like an arch, *Cicuta.*

— the back is bent forward, Ign.

**Biting** in back with chilliness, Lyc.

— in back, Agari, Kali-b., Thuja, Iris, *Graph.*, *Caust.*

**Blood.** Cannot lay on back on account of rush of blood to head, *Sul.*

— aching in small of back, preventing sleep, with orgasm of blood, with bruised sensation, *Sul.*

— boils on back, *Hepar*, Sul-a.

**Blotches.** Itching and red blotches on back, Asclep-t.

**Blow.** Heaviness and pressure in small of back, as if one had received a blow, worse while sitting, *Rhus.*

— pain in small of back, as from a blow, *Nux-m.*

— pain in back or coccyx, as from a blow or fall, or as if bruised, *Ruta.*

**Board.** Back painfully stiff like a board, *Puls.*

**Boils.** Small on back, neck, and arms, *Graph.*

— on back, Thuja, *Phyto.*, *Lach.*

— near small of back, with large red borders, Thuja.

— small suppurating boils on back, often leaving scar, *Kali-iod.*

— on back, near right side of spine, Kali-b.

— on back, with violent burning and throbbing, *Lach.*

**Boring** in back, *Bism.*, *Ham.*, Coccul.
— in a spot in back, Thuja.
— pressive pain in middle of back, Agari.
— pain in small of back, with a cold, Carbol-a.
**Bowels.** Pain in small of back if bowels did not move, *Cepa.*
— aching distress in bowels and back and legs with rumbling of wind, Ptelea.
**Break.** Pain in small of back, as if it would break, worse during rest, better by motion, *Kreos.*
— pain at the waist, as if she would break in two, runs up whole left side of body like lightning, seems as if they would give her a twist while it runs up with pain in elbow and wrist, *Corn.*
— pain in small of back, as if it would break, *Lyco.*
— pain in dorsal vertebræ, as if back would break, *Lil-tig.*
— pain as if back would break during menses, *Vib-op.*
— small of back feels as if it would break carrying paint, *Ham.*
— as if back would break just above waist, afterwards some pain higher up, Aletris.
— backache, as if it would break, *Bell.*
**Breakfast.** Complains very much of pain in lower back coming on after breakfast, but generally much increased by the monthly, Arg-n.
**Breaking.** Back feels as if breaking, *Nat-m.*, *Kalmia*, *Æsc-h.*, *Kali-c.*, Rhus, Variol., *Mag-c.*, Fer-iod.
— back feels as if breaking before stool, *Nux-v.*
— back feels as if breaking, with dysmenorrhœa, Xanthox.
**Breath.** Pain in back arresting the breath, Can-s.
— great restlessness, with yawning and stretching of arms and legs with pain in back, weariness as from too great exertion with awkward tottering gait and jerks, taking away the breath or going into abdomen, *Lach.*
— shifting pains in back, worse by drawing a long breath, Sang-c.

**Breath.** Stiffness now in back, now in hips, pains on turning over in bed, obliging her to hold the breath, Sul.
— pain all over back down to loins, worse on drawing a long breath, Cinnab.
— stinging, piercing, twitching in the back and sacrum, taking away his breath, Caustic.
— after falling from a height upon back acute pain in lower part, and in right side of back, especially when sneezing or quick breathing, Con.
— violent stitches in left side, above crest of ilium, more toward the back, arresting the breath, Mez.
— sudden hammering, better by pressure against a hard substance, sticking, tearing, so that he felt as if he would sink together and lose his breath, Sep.
— sticking at every breath when walking, itching, Sul.

**Breathe.** Violent pain in small of back, she can hardly breathe at beginning of menses, *Asar.*

**Breathing.** Pain goes up from small of back with stitches through chest and settled as stinging pains under left scapula, increased by deep breathing at 7 P. M., Zing.
— sprained pain in back and in scapula, extending into the chest, two or three times in a day, arresting the breathing, Petrol.
— stitches in small of back when coughing, breathing deeply, or walking, Arn.
— sticking upon breathing in small of back, Merc.
— stitching in side of back when breathing, Lyco.
— tearing in small of back, worse by breathing, Croc.
— when breathing frequent stitches from back toward chest, Psor.

**Broken,** drawing pain in small of back, and a feeling as if it was broken when walking, standing, and lying, *Carbo-a.*
— pain in small of back, as if broken, especially on touching, *Graph.*
— pain in back or small of back, as if bruised or broken, worse by pressure, Plat.

**Broken** pain in back, as if broken after walking an hour, Plat.
— pain in small of back, as if broken in pieces in morning in bed, Staph.
— violent pain, as if back were broken in lumbar region from least movement or coughing, or straining back from lifting, *Rhus.*
— pain as if back was broken, Nat-m., Variol.
— pain in back, as if broken in morning on stooping and on rising up, Ver-a.
— small of back pains, as if broken, Coral., *Nat-m.*
— pain in small of back, as if bruised or broken, worse at 3 or 4 A. M., *Nux-v.*
— pain in small of back, as if broken at night, *Mag-c.*
— intense pain in back and lumbar region, as if broken, *Eup-per.*
— small of back, as if broken, felt only at night, as if lying in a cramped position, Fer-iod.
— aching in back, as if bruised or broken, Convol.
— back aches, as if broken, *Kali-c.*
— small of back feels as if broken, *Ars.*
— as if broken in small of back with tension, worse when stooping, Clem.
**Bruise,** pain in back as from a bruise, EUP-PER.
— a pressive pain as from a bruise on lower part of back, with severe pressure in the scorbiculous cordis, same in rest as in motion, Coloc.
— pain as from a bruise in small of back second day of menses, Amm-c.
— pain as from a bruise or sprain in the whole back, with an inclination to stretch it, Agari.
**Bruised** feeling in back, ARN., Asar., Cornus., EUP-PER., Lyss., MERC., NUX-V., RHUS, Ruta, Sabad., Grat., Agari., Zinc., Berb., Stram., NAT-M., Nat-s., APIS, DROS., Vib-op., PHYTO., Kali-c., RAN-B., Ratan., Aco., Ars., Stront., GELS., Mag-s., NAT-C., Ox-a., PSOR., CHAM., MAG-M., Anac.

